# DNA–Polymer Nanostructures by RAFT Polymerization and Polymerization‐Induced Self‐Assembly

**DOI:** 10.1002/anie.201916177

**Published:** 2020-06-17

**Authors:** Thorsten Lückerath, Kaloian Koynov, Sebastian Loescher, Colette J. Whitfield, Lutz Nuhn, Andreas Walther, Christopher Barner‐Kowollik, David Y. W. Ng, Tanja Weil

**Affiliations:** ^1^ Synthesis of Macromolecules Max Planck Institute for Polymer Research Ackermannweg 10 55128 Mainz Germany; ^2^ Institute for Macromolecular Chemistry Freiburg University Stefan Meier Str. 31 79104 Freiburg Germany; ^3^ Freiburg Institute for Interactive Materials and Bioinspired Technologies (FIT) Georges-Köhler-Allee 105 79104 Freiburg Germany; ^4^ Centre for Materials Science, School of Chemistry and Physics Queensland University of Technology (QUT) 2 George Street QLD 4000 Brisbane Australia; ^5^ Macromolecular Architectures Institute for Chemical Technology and Polymer Chemistry (ITCP) Karlsruhe Institute of Technology (KIT) Engersserstraße 18 76131 Karlsruhe Germany

**Keywords:** DNA–polymer nanostructures, enzyme degassing, grafting-from approach, polymerization-induced self-assembly, RAFT polymerization

## Abstract

Nanostructures derived from amphiphilic DNA–polymer conjugates have emerged prominently due to their rich self‐assembly behavior; however, their synthesis is traditionally challenging. Here, we report a novel platform technology towards DNA–polymer nanostructures of various shapes by leveraging polymerization‐induced self‐assembly (PISA) for polymerization from single‐stranded DNA (ssDNA). A “grafting from” protocol for thermal RAFT polymerization from ssDNA under ambient conditions was developed and utilized for the synthesis of functional DNA–polymer conjugates and DNA–diblock conjugates derived from acrylates and acrylamides. Using this method, PISA was applied to manufacture isotropic and anisotropic DNA–polymer nanostructures by varying the chain length of the polymer block. The resulting nanostructures were further functionalized by hybridization with a dye‐labelled complementary ssDNA, thus establishing PISA as a powerful route towards intrinsically functional DNA–polymer nanostructures.

## Graphical abstract

**Pisa requirements**: Polymerization‐induced self‐assembly (PISA) is a novel platform technology for preparing intricate DNA–polymer nanostructures of various shapes by using RAFT polymerization directly from single‐stranded DNA. Due to the single‐stranded DNA shell, these nanostructures can be rationally functionalized by leveraging the complementarity of DNA. Thus, PISA offers a powerful pathway towards intrinsically functional DNA–polymer nanostructures.

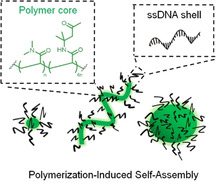

Polymerization reactions conducted directly on biomolecules have offered a unique access to complex bioconjugates with customizable polymer chain lengths and constituents.^1^ Although this notion implies that the polymerization would have to be accomplished under aqueous and mild conditions, it is assuring that modern radical polymerization techniques have progressed far to accommodate these requirements.^2^ As such, polymerization from biomolecules such as peptides,^3^ proteins,^4^ DNA/RNA,^5^ and even cel9ls^6^ has resulted in the generation of various functional biomolecule–polymer hybrid materials spanning multiple disciplines.^7^

Specifically, the combination of oligonucleotides with synthetic polymers has led to a series of hybrid materials with attractive applications as sensor devices^8^ or hydrogel drug delivery systems.^9^ Here, amphiphilic DNA–block copolymers, where a hydrophobic polymer is directly attached to DNA, have attracted significant attention due to their self‐assembly behaviors.^10^ Such assemblies were investigated as scaffolds for directing chemical reactions^11^ and for drug delivery applications.^12^ In addition, highly intricate 2D and 3D DNA nanoobjects were engineered via the DNA origami technology^13^ and served as templates to organize single polymer chains^14^ and to grow polymers in prescribed patterns with nanometer resolution.^15^ However, in many cases, the poor conjugation of DNA to the synthetic polymers is the major bottleneck, limiting their full potential.^16^

Hence, we envision that a “grafting from” approach will not only improve DNA–polymer coupling, but also provide access to nanostructures by leveraging polymerization‐induced self‐assembly (PISA).^17^, ^18^, ^19^ PISA on DNA has not been achieved previously due to compatibility issues between the DNA and the required polymerization conditions (i.e., ultralow reaction volume, high ionic strength, etc.). However, it is crucial to recognize that DNA brings forth a unique capability into PISA as DNA is intrinsically functional and can be post‐modified by the DNA hybridization technology. In this respect, while the first solution‐based RAFT polymerization from DNA was successful, it had to still rely on conventional degassing, which limited its robustness.^20^ Thus, we applied enzyme degassing as a significant progress for transferring polymerization processes to ultralow volumes and low radical concentrations.^21^, ^22^

In this way, the access to DNA–diblock copolymers of the type DNA‐A‐B (A and B denote different synthetic polymer units), as well as random DNA copolymers was made possible (Figure 1). Importantly, intrinsically functional DNA–polymer nanostructures were achieved for the first time via the PISA technique. Nanostructures such as micelles, worms, and vesicle‐like structures were formed based on RAFT polymerization from ssDNA and the growing polymer chains. We demonstrate post‐functionalization by DNA hybridization of the worm‐like structures, a unique property conferred by the oligonucleotide sequence. As such, the preparation of DNA–polymer nanostructures via the “grafting from” approach provides access to functional polymeric nanomaterials with complex shapes.

**Figure 1:**
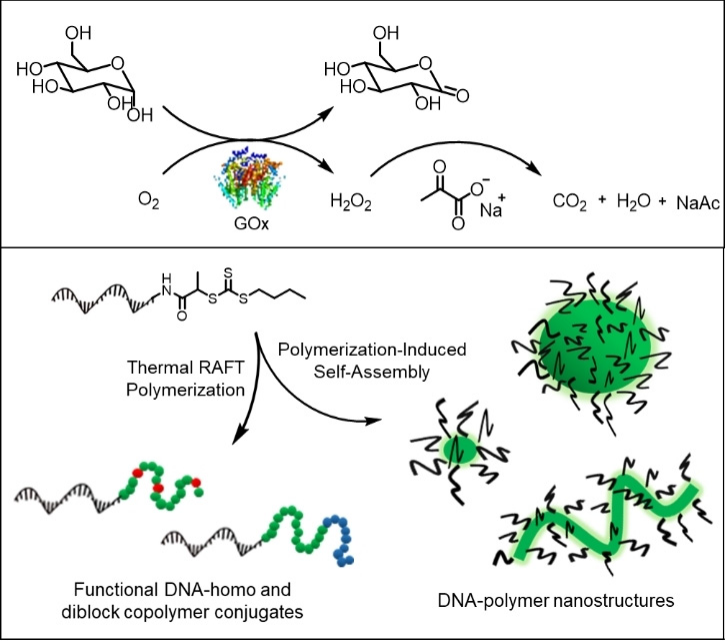
The concept for thermal RAFT polymerization from ssDNA under ambient conditions by using enzyme degassing is depicted. BTPA‐functionalized ssDNA served as the CTA in RAFT polymerization for the generation of functional DNA–homo and –diblock copolymer conjugates. Moreover, DNA–polymer nanostructures of various shapes were obtained by leveraging polymerization‐induced self‐assembly from ssDNA, establishing a new platform technology towards functional DNA–polymer nanostructures.

In order to synthesize the DNA–polymer conjugates, enzyme degassing was kept independent from the polymerization in line with other reports,^21^, ^23^ and the final concentrations were set to [glucose]=100 mm and [GOx]=1 μm. The polymerizations were conducted at 40 μL directly in a thermocycler (Figure S1) using the thermal initiator 2,2′‐azobis[2‐(2‐imidazolin‐2‐yl)propane] dihydrochloride (VA‐044). A critical drawback of enzyme degassing is that it converts oxygen into H_2_O_2_, which is reacts with dithiobenzoate‐ and trithiocarbonate‐based CTAs.^24^ Similar to the ATRP reported by Matyjaszewski and co‐workers,^25^ we added sodium pyruvate (SP) to minimize the effects of H_2_O_2_ during our RAFT polymerization.

These conditions were then transferred to the polymerization from 19‐mer ssDNA (3′‐ATC ATC CAC CAT CTC TTT T‐5′) equipped with a BTPA functionality (BTPA=2‐(*n*‐butyltrithiocarbonate) propionic acid) at its 5′ terminus (BTPA‐DNA), which was synthesized as published (Figure S2).^20^ The polymerizations from BTPA‐DNA using different monomers (*N*,*N*‐dimethylacrylamide (DMA), 4‐acryloylmorpholine (NAM), 2‐hydroxyethyl acrylate (HEA), oligo(ethylene glycol) methyl ether acrylate (OEGA)) and targeting different polymer lengths revealed narrow to moderate molecular weight distributions (*Đ*=1.14–1.41, Table S1) as determined by gel permeation chromatography (GPC) (Figure 2 a, Figure S3 a,b). The polymer lengths (*M*_n,app_=12.0–36.8 kDa) could be adjusted conveniently by altering the monomer to BTPA‐DNA ratio. The polymerizations were characterized by native polyacrylamide gel electrophoresis (PAGE) to demonstrate their efficiencies (Figure 2 b, Figure S3 c). Notably, minor leftover bands corresponding to BTPA‐DNA suggested that some end‐groups, ≈3–20 % depending on the monomer family, failed to initiate (Table S2). An in‐depth study on the BTPA stability by HPLC demonstrated that at least 50 mm of sodium pyruvate was necessary to achieve >90 % end‐group stability during the course of the polymerization (Figure S4 a,b). However, if a longer polymerization time is required, the amount of sodium pyruvate should be increased to maintain sufficient end‐group stability (Figure S4 c). In addition, independent HPLC and PAGE characterization proved that the DNA block remained intact during polymerization (Figures S5 and S6).

**Figure 2:**
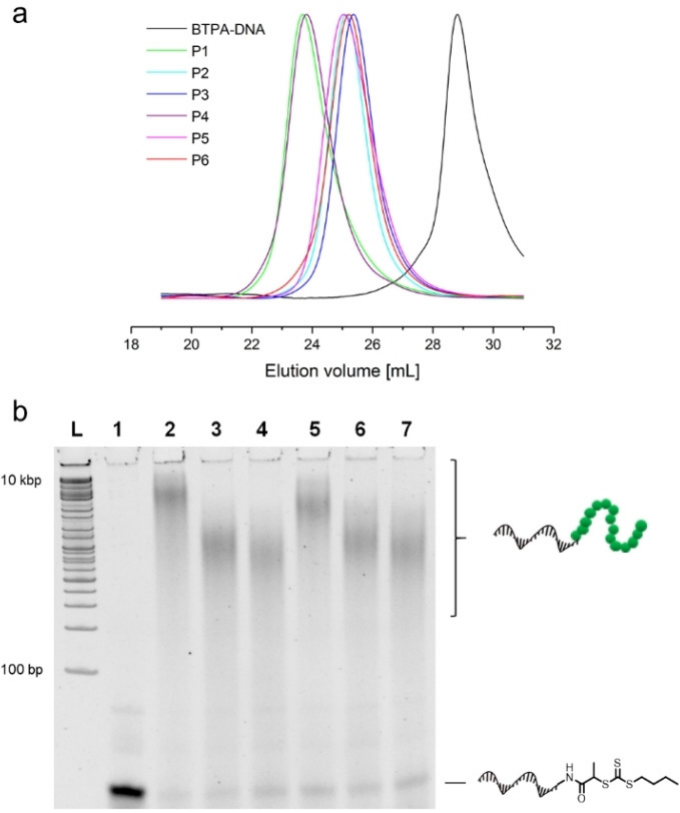
a) GPC traces of BTPA‐DNA (black line) and the DNA–polymer conjugates **P1**–**P6** (colored lines) as measured by DMF GPC using polymethylmethacrylate (PMMA) calibration standards. b) BTPA‐DNA and the DNA–polymer conjugates **P1**–**P6** analyzed by 20 % native PAGE. L: DNA ladder; lane 1: BTPA‐DNA; lanes 2–7: **P1**–**P6** (from left to right).

The DNA functionality for hybridization was probed by fluorescence correlation spectroscopy (FCS)^26^ using a rhodamine 6G complementary DNA strand (3′‐GAG ATG GTG GAT GAT TTT T‐5′) (Figure S3 d). A series of additional control studies to exclude nonspecific adsorption or entanglement of the DNA to the polymer was accomplished using PAGE (Figure S7). Further characterization was attempted by copolymerizing HEA with a rhodamine B containing acrylate, affording the rhodamine B containing DNA–polymer conjugate **FP1**. The successful incorporation of rhodamine B into the polymer block was confirmed by FCS (Figure 3 b) and the copolymerization proceeded with good control (*Đ*=1.27, Table S4, Figure S8). Complementarity and specificity was accomplished using FRET. Therefore, **FP1** was hybridized with a complementary 19mer sequence (3′‐GAG ATG GTG GAT GAT TTT T‐5′) carrying Cy5 at its 3′ terminus (Figure 3 a). As a control, a mismatched sequence was used. Acceptor emission of Cy5 showed a clear FRET and a significantly greater intensity compared to the mismatched sequence (Figure 3 c, Figure S9). The above method was expanded to conduct the first block copolymerization on DNA via the “grafting from” approach. Polymerizations from BTPA‐DNA with first DMA followed by NAM were performed. Therefore, the added solution of NAM had to be supplemented with the enzyme degassing system, otherwise, the polymerization of the second block would stop at low conversions (Table S5, Figure S5 a,b). The GPC traces revealed clear shifts towards higher molecular weights, while narrow molecular weight distributions were maintained with high end‐group fidelity (Figure 3 e, Figure S10). The growth of each polymer block was additionally monitored by PAGE (Figure 3 d), confirming the successful synthesis of ssDNA–diblock copolymers of the type ssDNA‐*b*‐A‐*b*‐B, with A and B denoting different synthetic polymer units, by the grafting‐from approach.

**Figure 3:**
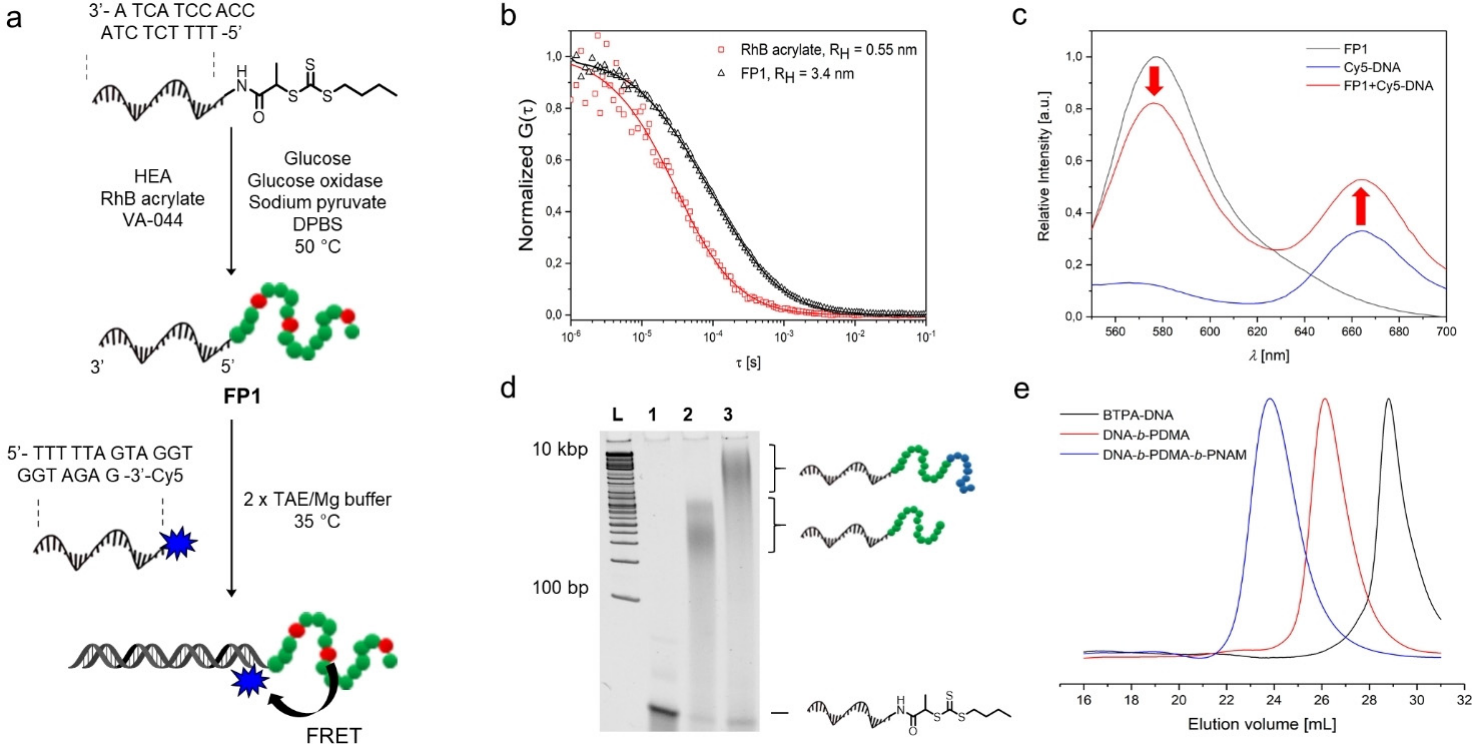
a) Schematic representation of the synthesis of a rhodamine B containing DNA–polymer conjugate (**FP1**) and its subsequent labelling with a complementary DNA sequence containing Cy5 at its 3′‐terminus. b) Normalized FCS autocorrelation curves measured in aqueous solutions of rhodamine B acrylate (red symbols) and **FP1** (black symbols). The solid lines represent the corresponding fit with Equation S1, which yielded the hydrodynamic radii of rhodamine B acrylate (*R*_H_=0.55 nm) and **FP1** (*R*_H_=3.4 nm). c) Emission spectra of **FP1** alone (black line), Cy5‐DNA (blue line) and **FP1** hybridized with Cy5‐DNA (red line) upon excitation at 485/20 nm. d) Monitoring of the block copolymerization from DNA by 20 % native PAGE. L: DNA ladder; lane 1: BTPA‐DNA; lane 2: DNA‐*b*‐PDMA; lane 3: DNA‐*b*‐PDMA‐*b*‐PNAM. e) GPC traces of BTPA‐DNA (black line), DNA‐*b*‐PDMA (red line), and DNA‐*b*‐PDMA‐*b*‐PNAM (blue line) as measured by DMF GPC using PMMA calibration standards.

PISA by using RAFT polymerization has emerged prominently for the preparation of intricate block copolymer assemblies.^18^, ^19^ With increasing degrees of polymerization, the structures reassemble into the thermodynamically most favored state, enabling structural control by targeting different chain lengths (Figure 4 a). Diacetone acrylamide (DAAm) and dimethylacrylamide (DMA) were selected for conducting PISA from DNA according to PISA based purely on synthetic polymers.^27^ Here, a constant [DAAm]/[DMA] ratio of 80:20 was applied and different polymer chain lengths (DP_n_=50, 100, 200, and 250) were envisaged. The polymerizations were conducted in Dulbecco's Phosphate Buffered Saline (DPBS) at >90 % conversion (Table S6). GPC analysis in a non‐selective solvent (i.e., DMF) revealed that well‐controlled polymerizations were maintained during the PISA process (Figure S11).

**Figure 4:**
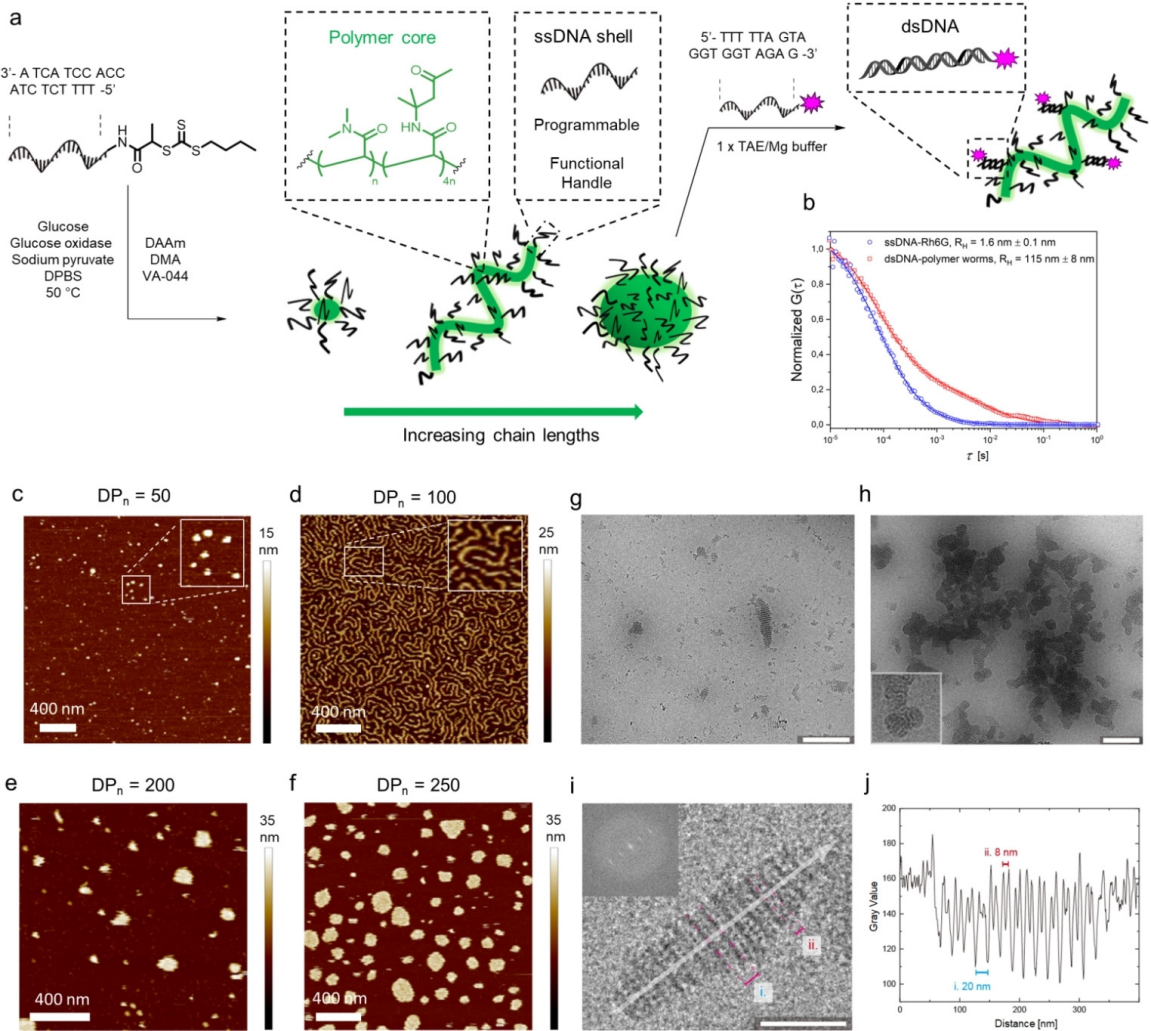
a) Schematic representation of PISA from DNA using DMA and DAAm as the monomers for chain‐extension from DNA. The resulting DNA–polymer nanostructures can be further functionalized by hybridization of a functional complementary DNA sequence to the available DNA ends of the nanostructures. b) Normalized FCS autocorrelation curves measured in aqueous solutions of rhodamine 6G‐DNA (red symbols) and the DNA–polymer worms hybridized with rhodamine 6G‐DNA (blue symbols). The solid lines represent the corresponding fit with Equation S1, which yielded the hydrodynamic radii of the rhodamine 6G‐DNA (*R*_H_=1.6 nm) and the functionalized DNA–polymer worms (*R*_H_=115 nm). c–f) AFM images recorded by liquid AFM after aqueous RAFT dispersion polymerization from BTPA‐DNA using a [DAAm]/[DMA] ratio of 80:20. Different degrees of polymerization were targeted: DP_n_=50 (c), 100 (d), 200 (e), 250 (f). The magnified images in (b) and (c) are 2.5 times magnified with respect to the original picture. g) Cryo‐TEM images of DNA–polymer worms (DP_n_=100) at a concentration of 80 μm. h) Cryo‐TEM images of DNA–polymer disc assemblies (DP_n_=200) at a concentration of 400 μm. (i,j) Gray‐scale analysis of DNA–polymer worms (DP_n_=100). The inset shows the fast Fourier transformation of the highly ordered structure. The gray‐scale plot along the longer axis (indicated by the arrow) shows the periodicity of the structural features and their distances from each other.

The resulting nanostructures were visualized at 4 μm by liquid atomic force microscopy (AFM) from a Mg^2+^‐containing 1×TRIS‐acetate‐EDTA (TAE) buffer.^28^ When low degrees of polymerization (i.e., DP_n_=50) were targeted, spherical micelle‐like structures with a mean height of 12 nm were formed (Figure 4 c, Figures S12 and S16 a). A morphology transition from micelles to worm‐like structures was observed upon increasing the polymer length from DP_n_=50 to DP_n_=100 (Figure 4 d, Figure S13). These worm‐like structures exhibited similar mean heights as the micellar structures (14 nm, Figure S16 b) and their lengths varied from 100 nm to 1 μm. With higher degrees of polymerization (DP_n_=200 and 250), the increased polymer length caused the worms to reorganize into larger nanostructures with variable lateral sizes up to several hundred nanometers (Figure 4 e,f, Figures S14 and S15). Interestingly, these structures instead displayed characteristically homogenous mean heights of about 18 nm (Figure S16 c,d). Further characterization was accomplished via dynamic light scattering (DLS) at 10 μm, where size distributions corresponding to the micelles (16.5±1.5 nm), worms (27.3±0.6 nm), and disc‐like aggregates (53.7±0.9) were observed (Figure S17).

Cryo‐TEM measurements of vitrified DNA–polymer dispersions were conducted to assess the particle structures in solution at higher concentrations. At such concentrations, larger substructured particles of the DNA‐polymer nanostructures were observed, being highly reminiscent of so‐called inverse morphologies.^29^ For instance, the DNA–polymer worm assemblies (DP_n_=100, *c*=80 μm) showed a large abundance of striped multicompartment particles that were up to 300 nm in length (Figure 4 g,i). These particles were most likely composed of layers of cylinders packed in alternating layers rotated by 90°. Layers in plane with the imaging plane appeared as solid lines and were spaced by roughly 20 nm (Figure 4 j). The interstitial space was substructured with cylinders perpendicular to the imaging plane. The sample that was identified as disc‐like aggregates (DP_n_=200) in AFM formed multicompartment aggregates at higher concentrations (*c*=400 μm), which were composed of patches of worm‐like nanostructures (Figure 4 h).

The observation of these different morphologies is reasonable considering that such inverse morphologies typically occur at lower solubility, i.e., in the presence of short DNA blocks and higher ionic strength. The different morphologies observed in AFM and cryo‐TEM demonstrate the unique influence of the DNA block towards the assemblies at different concentrations.

We selected the worm‐like nanostructure to demonstrate that such sensitive structures could be functionalized using the DNA hybridization technology. Rhodamine 6G labelled complementary ssDNA′ was hybridized onto the PISA worms and characterized via DLS, AFM, and FCS. On DLS, the size of the PISA worms did not change upon hybridization compared to the control (Figure S18). FCS monitoring of the rhodamine 6G DNA clearly indicated that DNA was indeed hybridized due to an increase of the hydrodynamic radius from 1.6 nm to 115 nm (Figure 4 b), implying successful attachment of the complementary DNA. A two‐component fit of the autocorrelation curve indicated that ≈20 % hybridization was achieved using a 1:10 mol ratio of Rho6G‐ssDNA′ to the ssDNA–polymer chains. AFM visualization of the hybridized PISA worms showed moderate deformation of the worms while still maintaining elongated morphologies (Figure S19).

In conclusion, we have introduced the first solution‐based thermal RAFT polymerization from DNA under ambient conditions by relying on enzyme degassing with glucose, glucose oxidase, and sodium pyruvate. A series of DNA–polymer conjugates derived from acrylamide (DMA, NAM) and acrylate (HEA, OEGA) monomers as well as DNA–diblock copolymers were synthesized with narrow molecular weight distributions and varying lengths. Crucially, we performed for the first time PISA with RAFT polymerization from DNA, thus providing a convenient route for the construction of complex DNA–polymer architectures such as micelles or worms. With the current achievements in PISA of block copolymers consisting purely of synthetic polymers, we envisage that the combination of PISA with DNA will have a major impact on DNA nanotechnology and polymer nanostructuring.

## Conflict of interest

The authors declare no conflict of interest.

## Supporting information

As a service to our authors and readers, this journal provides supporting information supplied by the authors. Such materials are peer reviewed and may be re‐organized for online delivery, but are not copy‐edited or typeset. Technical support issues arising from supporting information (other than missing files) should be addressed to the authors.

Supplementary
