# Supplementary material for: DNA–Polymer Nanostructures by RAFT Polymerization and Polymerization‐Induced Self‐Assembly
Source: Angew Chem Int Ed Engl. 2020 Jun 17;59(36):15474–9. doi: 10.1002/anie.201916177 (PMC7496909; doi:10.1002/anie.201916177)
Supplement: Supplementary file 1 — Supplementary [file ANIE-59-15474-s001.pdf]

Angewandte Chemie
Eine Zeitschrift der Gesellschaft Deutscher Chemiker

Supporting Information

# DNA–Polymer Nanostructures by RAFT Polymerization and Polymerization-Induced Self-Assembly

*Thorsten Lückerath, Kaloian Koynov, Sebastian Loescher, Colette J. Whitfield, Lutz Nuhn, Andreas Walther, Christopher Barner-Kowollik, David Y. W. Ng,* and Tanja Weil**

anie_201916177_sm_miscellaneous_information.pdf

## Table of Contents

**Experimental Procedures** p.3

- Chemicals
- Methods
  - Ultraviolet-Visible (UV-VIS) Spectroscopy)
  - Gel Permeation Chromatography (GPC)
  - Matrix-Assisted Laser Desorption/ionization-Time of Flight (MALDI-ToF) Mass Spectrometry
  - Polyacrylamide Gel Electrophoresis (PAGE)
  - High-Performance Liquid Chromatography (HPLC)
  - Fluorescence Correlation Spectroscopy (FCS)
  - Atomic Force Microscopy (AFM)
  - Dynamic Light Scattering (DLS)
- Experimental Procedures

**Results and Discussion** p.6

- Figure S1
- Figure S2
- Table S1
- Figure S3
- Table S2
- Figure S4
- Table S2
- Figure S5
- Figure S6
- Table S3
- Figure S7
- Table S4
- Figure S8
- Figure S9
- Table S5
- Figure S10
- Table S6
- Figure S11
- Figure S12
- Figure S13
- Figure S14
- Figure S15

Figure S16

Figure S17

Figure S18

Figure S19

**References** **p.17**

**Author Contributions** **p.17**

## Experimental Procedures

### Chemicals

D-Glucose (Sigma), sodium pyruvate (SP, > 99%, Carl Roth) and acryloxyethyl thiocarbamaoyl rhodamine B (RA, Polysciences, Inc.) were used as received. Glucose oxidase from *Aspergillus niger* (GOx, Sigma, 100 000–250 000 U/g) was dissolved in Dulbecco's Phosphate Buffered Saline (DPBS, Sigma, pH = 6) and stored in aliquots at −20 °C. All functional single-stranded DNAs used within this study were purchased from biomers.net. BTPA-terminated DNA was synthesized from amine-terminated DNA as was previously reported.[1] The TAE/Mg buffers used within this study were diluted from a 20 × stock solution containing 20 mM $Na_2EDTA$, 100 mM NaCl, 100 mM TRIS and 240 mM $MgCl_2$ * 6 $H_2O$. Dulbecco's Phosphate Buffered Saline was purchased from Sigma Aldrich.

Poly(ethylene glycol) methyl ether acrylate (OEGA, Sigma, $M_n$ = 480 g/mol, stabilized with 100 ppm MeHQ and 100 ppm BHT), 2-hydroxyethyl acrylate (HEA, Sigma, 96%, stabilized with 200–650 ppm MEHQ), *N*,*N*-dimethylacrylamide (DMA, Acros Organics, stabilized with 500 ppm MeHQ) and 4-acryloylmorpholine (NAM, Sigma, 97%, stabilized with 1000 ppm MEHQ) were passed through a short column of alumina prior to use and were stored at 4 °C. Diacetone acrylamide (DAAm, Sigma, 99%) was recrystallized from cyclohexane once prior to use. 2,2'-Azobis[2-(2-imidazolin-2-yl)propane] dihydrochloride (VA-044, FluoroChem Ltd.) was recrystallized from methanol once prior to use.

### Methods

### Ultraviolet-Visible (UV-VIS) Spectroscopy)

UV-VIS spectra were recorded on a Spark ® 20M from Tecan Group Ltd. using either a NanoQuant PlateTM or a 384 well UV-Star microplate.

The sample preparation for FRET measurements was conducted by hybridizing Cy5-DNA (c = 10 $\mu$M) to the rhodamine B-containing DNA-polymer conjugate **FP1** (c = 10 $\mu$M) by incubation in 2 × TAE/Mg buffer at 37 °C for 2 h. Cy5-DNA as well as the rhodamine B-containing DNA-polymer conjugate **FP1** were incubated alone at identical conditions to account for possible photobleaching and served as reference samples. For UV-VIS measurements, each sample was diluted to 200 nM and was excited using an excitation filter at 485/20 nm.

### Gel Permeation Chromatography (GPC)

GPC experiments were performed on a PSS SECcurity instrument comprising an autosampler, a column oven with 3 GRAM columns ($10^3$, $10^3$ and $10^2$ Å, 300 × 8 mm, 10 $\mu$m particle size) and a RI as well as an UV detector (Agilent Technologies 1260 Infinity). DMF containing 1g/L lithium bromide was used as the eluent at a flowrate of 1 mL/min. Poly(methyl methacrylate) (1600 kDa–800 Da) served as the calibration standard for molecular weight measurements. The samples were filtered (0.4 µm) prior to injection. The data was processed with the software PSS WINGPC UniChrom.

### Matrix-Assisted Laser Desorption/ionization-Time of Flight (MALDI-ToF) Mass Spectrometry

MALDI-ToF mass spectra were acquired on a rapiflexTM MALDI-ToF/ToF mass spectrometer from Bruker equipped with a 10 kHz scanning smartbeam 3D laser (Nd:YAG at 355 nm) and a 10 bit 5 GHz digitizer. Measurements were performed in the positive reflector mode using 3-hydroxypicolinic acid as the matrix. The samples were prepared by incubating a solution of 1 µL of DNA sample (100 $\mu$M in DNA) and 2 $\mu$L of matrix solution (95 $\mu$L of 3-hydroxypicolinic acid in acetonitrile/water (1:1, 50 g/L) + 10 $\mu$L of ammonium citrate dibasic in water (100 g/L)) with an ion exchange resin. The samples were then applied to the target plate and were left for crystallization. Prior to every measurement, the instrument was calibrated using 3 commercially purchased DNAs (3648 Da, 6120 Da, 9195 Da). The data was processed with mMass.

### Polyacrylamide Gel Electrophoresis (PAGE)

Native PAGE was performed on 10 % or 20 % NovexTM Tris-Borate-EDTA (TBE) gels (12 wells, Thermo Fisher). The gels were run on a Cell SureLockTM mini-cell electrophoresis system from Thermo Fisher using 0.5 × TBE buffer as the running buffer (44.5 mM Tris-Borate, 1 mM EDTA).

Electrophoresis was conducted at 175 V for 90 min (20 % gels) or at 135 V for 50 min (10 % gels). Each sample was prepared by first hybridizing 1 $\mu$L DNA sample (100 $\mu$M, 100 pmol) with the complementary Rh6G-DNA sequence (100 $\mu$M, 50 pmol) in 3.5 $\mu$L 0.5 × TBE buffer at 35 °C for 30 min in order to increase gel resolution as reported previously.[2] 1 $\mu$L DNA gel loading dye (6 × Thermo Fisher) was then added prior to running the gel. GeneRuler low Range DNA ladder (Thermo Fisher) was used as received. Gels were subsequently stained with SYBR Gold in 50 mL 0.5 × TBE buffer for 30 min at room temperature.

Purification of the polymers was accomplished by PAGE extraction as previously published.[3]

### High-Performance Liquid Chromatography (HPLC)

Measurements were performed on a HPLC instrument from Shimadzu comprising an auto sampler, a column oven and a fraction collector. The samples were purified either by semi-prep HPLC using a ZORBAX Eclipse XDB-C18 HPLC column (9.4 × 250 mm, 5 $\mu$m) from Agilent at a flowrate of 4 mL/min or by analytical HPLC using the same column type (4.6 × 250 mm, 5 $\mu$m) and the identical elution protocol at a flowrate of 1 mL/min. The elution protocol started with the mobile phase from 5% solvent B (HPLC grade acetonitrile) and 95% solvent C (0.1 M triethylammonium acetate buffer), raising linearly first to 60 % B in 20 min, then to 100% B in 3 min, then decreasing to 5% B in 4 min and finally holding 5% B for 3 min. The absorbance was monitored at 310 nm and 254 nm.

### Fluorescence Correlation Spectroscopy (FCS)

FCS experiments were performed on a commercial setup (Carl Zeiss, Germany) consisting of the module Confocor 2 and an inverted microscope Axiovert 200 using a C-Apochromat 40×/1.2W water immersion objective. The excitation was done by the 543 nm line of a HeNe laser and the collected fluorescence signal was filtered through a LP560 long pass emission filter before reaching the detector, an avalanche photodiode that enables single-photon counting. An eight-well, polystyrene chambered cover-glass (Lab-Tek, Nalge Nunc International) was used as a sample cell. For each solution, a series of five measurements with a total duration of five min were performed. The confocal observation volume was calibrated using a reference dye with known diffusion coefficients, i.e. Alexa 546. The experimentally measured autocorrelation curves were fitted with the model function for an ensemble of *m* different types of freely diffusing fluorescent species:

$$G(\tau) = 1 + \frac{1}{N}\sum_{i=1}^{m} \frac{f_i}{\left(1+\frac{\tau}{\tau_{D,i}}\right)\cdot\sqrt{1+\frac{\tau}{S^2\cdot\tau_{D,i}}}} \qquad \text{(S1)}$$

Here, *N* is the average number of diffusing fluorescent species in the observation volume, $\tau_{D,i}$ is the diffusion time of the *i*-th species, $f_i$ is the fraction of component *i* and *S* is the so-called structure parameter $S = \frac{z_0}{r_0}$, where $z_0$ and $r_0$ represent the axial and radial dimension of the confocal volume, respectively. Furthermore, the diffusion time $\tau_{D,i}$ is related to the respective diffusion coefficient $D_i$, through $D_i = \frac{r_0^2}{4\cdot\tau_{D,i}}$. From the fits, the diffusion times and consecutively the diffusion coefficients of the fluorescent species were determined. Finally, the hydrodynamic radii $R_H$ of the fluorescent species were calculated through the Stokes-Einstein relation $R_H = k_B T / 6\pi\eta D$ where *T* is the temperature, $k_B$ the Boltzmann constant and $\eta$ the viscosity of water.

Samples of nonfunctional DNA-polymer conjugates were prepared by first hybridizing the complementary rhodamine 6G-containing DNA strand in 2 × TAE/Mg buffer at 37 °C for 1 hour ([DNA-polymer] = 20 $\mu$M , [Rho6G-DNA] = 10 $\mu$M) and then diluting to a final rhodamine concentration of ~ 50 nM.

Samples of rhodamine B-containing DNA-polymer conjugates were prepared by just diluting to a final dye concentration of ~ 50 nM.

PISA samples were prepared by hybridizing the complementary Rh6G-containing DNA sequence to the DNA-polymer aggregates in 1 × TAE/Mg buffer at room temperature for 15 min (Final concentration: [DNA-polymer] = 25 µM, [Rho6G-DNA] = 2.5 µM). The measurements were conducted at higher fluorophore concentrations ([Rh6G] > 1 $\mu$M) to ensure stability of the DNA-polymer nanostructures.

In all cases, the experimental autocorrelation curves were fitted with two component models (m=2 in eq. S1) to account for the small amounts of freely diffusing Rho6G remaining from the ssDNA labelling or rhodamine B-containing monomer remaining after purification.

### Atomic Force Microscopy (AFM)

AFM measurements were conducted on a Dimension FastScan Bio™ atomic force microscope from Bruker, which was operated in the PeakForce mode. AFM probes with a nominal spring constant of 0.25 $Nm^{-1}$ were employed (FastScan-D, Bruker) for measurement in liquid. A circular mica disc (15 mm) was used as the substrate. Measurements were performed at scan rates

between 0.8 and 2 Hz. Different areas of the mica substrate were scanned in order to ensure the integrity of the shown images. The images were finally processed by the software NanoScope Analysis 1.8.

For sample preparation, 50 μL 1 × TAE/Mg buffer (12 mM $Mg^{2+}$) containing the DNA-polymer nanostructure at [DNA] = 4 μM was applied onto a freshly cleaved mica substrate. The solution was left to incubate for 10 min in order to deposit the desired species on the mica surface. After successful adsorption, the supernatant was removed and fresh 1 × TAE/Mg buffer (200 μL) was added for the measurement.

### Dynamic Light Scattering (DLS)

Dynamic light scattering (DLS) were recorded at 25 °C on a Zetasizer Nano S (Malvern Instruments Ltd, Malvern, U.K.) equipped with a HeNe laser (λ = 633 nm) and detected at a scattering angle of 173°. All measurements were performed in triplicate. The obtained data was processed by cumulant fitting for z-average and PDI, or by CONTIN fitting for intensity/ volume/ number weighted particle size distribution.

Samples were prepared at ~ [DNA] = 10 μM in 1 × TAE/Mg buffer and dust could be removed from the sample prior to each measurement by filtration through a GHP syringe filter (0.22 μm pore size, Acrodisc).

### Cryogenic Transmission Electron Microscopy (Cryo-TEM)

Cryo-TEM measurements were performed on a FEI Talos 120C electron microscope at 120 kV operating voltage. Cryo-TEM samples were prepared on holey carbon films with various hole sizes using a FEI Vitrobot Mk IV. The samples were freshly prepared in $Mg^{2+}$-containing (12 mM) 1 × TAE buffer prior to the measurements. Image analysis was performed with the Fiji plug-in version of Imagej.

### Experimental Procedures

DPBS was adjusted to pH = 6 with diluted HCl prior to the experiments. Stock solutions were prepared of BTPA (20 mM), VA-044 (2 mM) and rhodamine acrylate (25 mM) in *t*-butanol/DPBS (30/70, v/v) as well as BTPA-DNA (10 mM), glucose (0.8 M), glucose oxidase (8 μM), sodium pyruvate (2 M), DAAm (2 M) and DMA (500 mM) in pure DPBS.

For a typical polymerization, monomer, BTPA stock, VA-044 stock, sodium pyruvate stock (1.25 μL) and DMF (1.25 μL) were diluted with DPBS and *t*-butanol/DPBS (30/70, v/v) to a total volume of 37.5 μL and to a solvent composition of *t*-butanol/DPBS (20/80, v/v). The amounts of monomer, BTPA stock and VA-044 stock were varied according to the employed concentrations. To this mixture glucose stock (6.25 μL) and glucose oxidase stock (6.25 μL) were added, resulting in a total volume of 50 μL in *t*-butanol/DPBS (15/85, v/v). 40 μL of this solution was pipetted into a PCR tube, which was closed and placed in a thermocycler at the appropriate temperature. The remaining solution served as $t_0$ sample for the determination of the conversion. At the completion of the polymerization, 15 μL were diluted in DMF for GPC and another 15 μL were diluted in $D_2O$ for measuring the conversion via $^1H$ NMR spectroscopy.

For the synthesis of DNA-*b*-polymer conjugates, the identical protocol was pursued with the exception that BTPA-DNA stock was used instead of BTPA stock.

DNA-diblock copolymer conjugates were achieved by removing 20 μL of the reaction mixture after polymerization of the first block and adding 20 μL of freshly prepared solution containing the monomer of the second block at the appropriate concentration as well as glucose at 100 mM, glucose oxidase at 1 μM and sodium pyruvate at 50 mM.

For the synthesis of rhodamine B-containing DNA-polymer conjugates, the general polymerization protocol was followed with the exception that RA stock was additionally added at the appropriate concentration.

For the PISA experiments, the general polymerization protocol was followed with the exception that DAAm and DMA stocks were added at the appropriate concentrations. Also, the solvent was changed to pure DPBS by preparing VA-044 stock (2 mM) in DPBS instead of *t*-butanol/DPBS (30/70, v/v).

## Results and Discussion

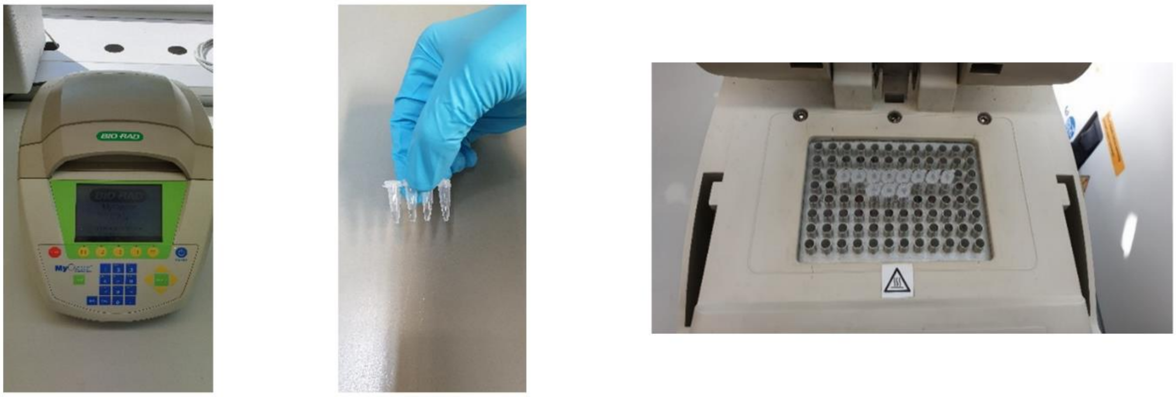

**Figure S1**. Pictures of the employed thermocycler (BioRad MyCycler Thermal Cycler, left), PCR tubes filled with 40 $\mu$L reaction volume (middle) and PCR tubes placed in the thermocycler for conducting the polymerizations (right).

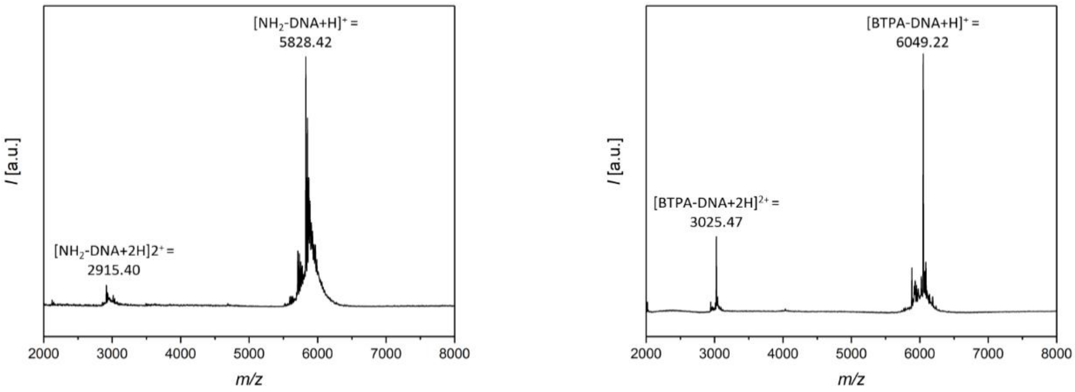


**Figure S2**. Mass spectra of $NH_2$-DNA (left, mass calculated: 5828.89 Da) and BTPA-DNA after HPLC purification (right, mass calculated: 6049.48 Da)

**Table S1.** Homopolymerization from BTPA-DNA via thermal RAFT polymerization under ambient conditions.

| Entry | Monomer | [M]:[BTPA-DNA]:[I] [a] | Time (h) | [BTPA] (mM) | Conversion [b] (%) | $M_{n,\ theo.}$ [c] (kDa) | $M_{n,\ app.}$ [d] (kDa) | $Đ$ [d] |
|---|---|---|---|---|---|---|---|---|
| P1 | DMA | 500:1:0.1 | 6 | 1 | 90 | 50.7 | 36.8 | 1.24 |
| P2 | DMA | 375:1:0.1 | 4 | 1 | 64 | 29.8 | 23.2 | 1.14 |
| P3 | DMA | 250:1:0.1 | 4 | 1 | 91 | 28.6 | 20.0 | 1.19 |
| P4 | NAM | 500:1:0.1 | 4 | 1 | 72 | 56.9 | 35.2 | 1.33 |
| P5 | NAM | 375:1:0.1 | 4 | 1 | 60 | 37.8 | 20.1 | 1.28 |
| P6 | NAM | 250:1:0.1 | 4 | 1 | 65 | 29.0 | 20.6 | 1.32 |
| P7 | HEA | 250:1:0.1 | 4 | 2 | 79 | 29.0 | 29.9 | 1.23 |
| P8 | HEA | 125:1:0.1 | 4 | 2 | 61 | 14.9 | 12.6 | 1.27 |
| P9 | OEGA | 80:1:0.1 | 4 | 1 | 51 | 25.6 | 14.2 | 1.41 |
| P10 | OEGA | 60:1:0.1 | 4 | 1 | 51 | 20.7 | 12.0 | 1.34 |

[a] Polymerizations were conducted at 45 °C in DPBS/*t*BuOH (85/15, v/v) using enzyme degassing at [glucose] = 100 mM, [GOx] = 1 $\mu$M and [SP] = 50 mM. [b] Conversions were determined by $^1$H NMR in $D_2O$ using DMF (2.5 %) as internal standard. [c] Theoretical molecular weights were calculated based on the following equation: $M_{n,\ theo.} = [M]_0/[DNA]_0 \times MW^M \times conv.(NMR) + MW^{DNA}$. [d] Apparent molecular weights and dispersity values were determined by GPC with DMF as the eluent using PMMA calibration standards without correction.

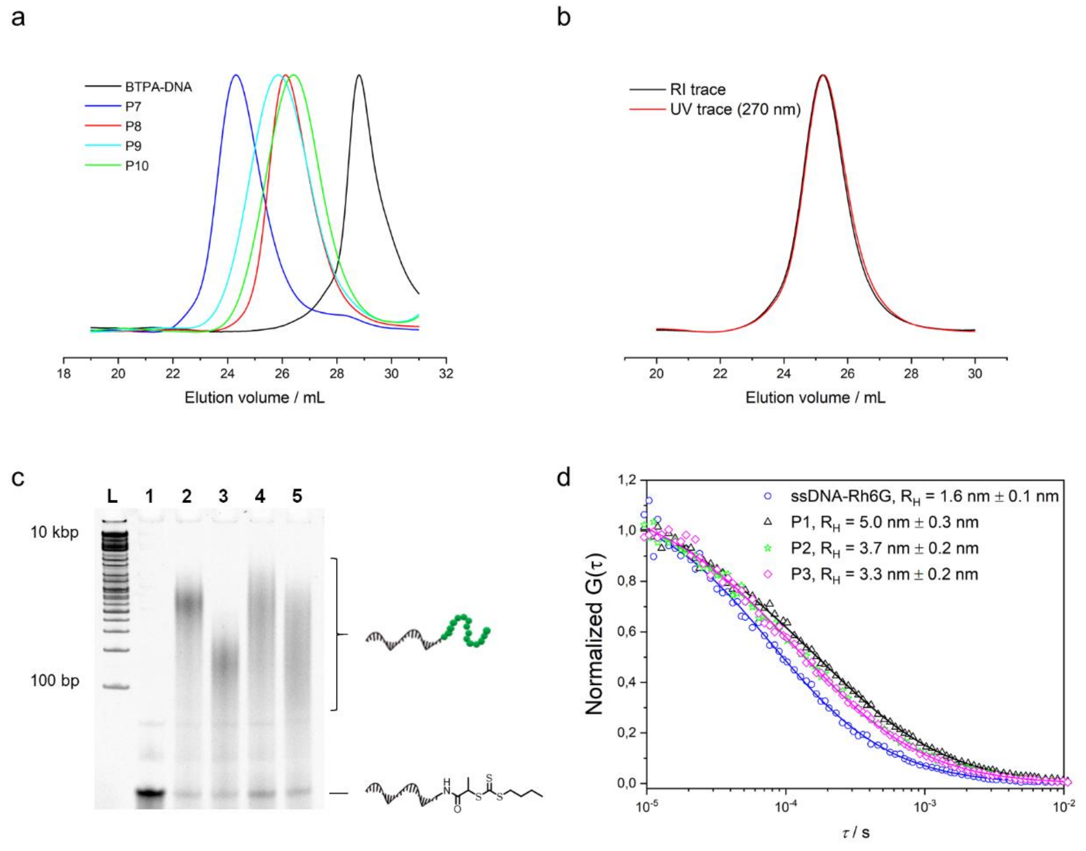


**Figure S3.** (a**)** GPC traces of BTPA-DNA (black line) and the DNA-polymer conjugates **P7-P10** (colored lines) as measured by DMF GPC with PMMA calibration standards. (b) Overlay of the RI trace (black line) and the UV trace at λ = 270 nm (red line) after polymerization from DNA as measured by DMF GPC using PMMA calibration standards. (c) BTPA-DNA and the DNA-polymer conjugates **P7-P10** analyzed by 20% native PAGE. L, DNA ladder; lane 1, BTPA-DNA; lane 2-5, **P7-P10** (from left to right). (d) Normalized FCS autocorrelation curves (symbols) measured in aqueous solutions of rhodamine 6G-terminated DNA and DNA-polymer conjugates **P1**–**P3** hybridized with it. The solid lines represent the corresponding fits with eq. S1, which yielded the hydrodynamic radii of rhodamine 6G-terminated DNA ($R_H$ = 1.6 nm), **P1** ($R_H$ = 3.3 nm), **P2** ($R_H$ = 3.7 nm) and **P3** ($R_H$ = 5.0 nm).

**Table S2.** Overview on the percentage of left-over DNA after DNA polymerization as calculated by Image J[4] from the PAGE gels.

| Entry | Monomer | [M]:[BTPA-DNA]:[I] | Remaining DNA [%] | DNA-Polymer Conjugate [%] |
|---|---|---|---|---|
| P1 | DMA | 500:1:0.1 | 3.0 | 97.0 |
| P2 | DMA | 375:1:0.1 | 4.0 | 96.0 |
| P3 | DMA | 250:1:0.1 | 5.7 | 94.3 |
| P4 | NAM | 500:1:0.1 | 6.4 | 93.6 |
| P5 | NAM | 375:1:0.1 | 6.5 | 93.5 |
| P6 | NAM | 250:1:0.1 | 7.5 | 92.5 |
| P7 | HEA | 250:1:0.1 | 8.2 | 91.8 |
| P8 | HEA | 125:1:0.1 | 13.1 | 86.9 |
| P9 | OEGA | 80:1:0.1 | 23.4 | 76.6 |
| P10 | OEGA | 60:1:0.1 | 19.6 | 80.4 |

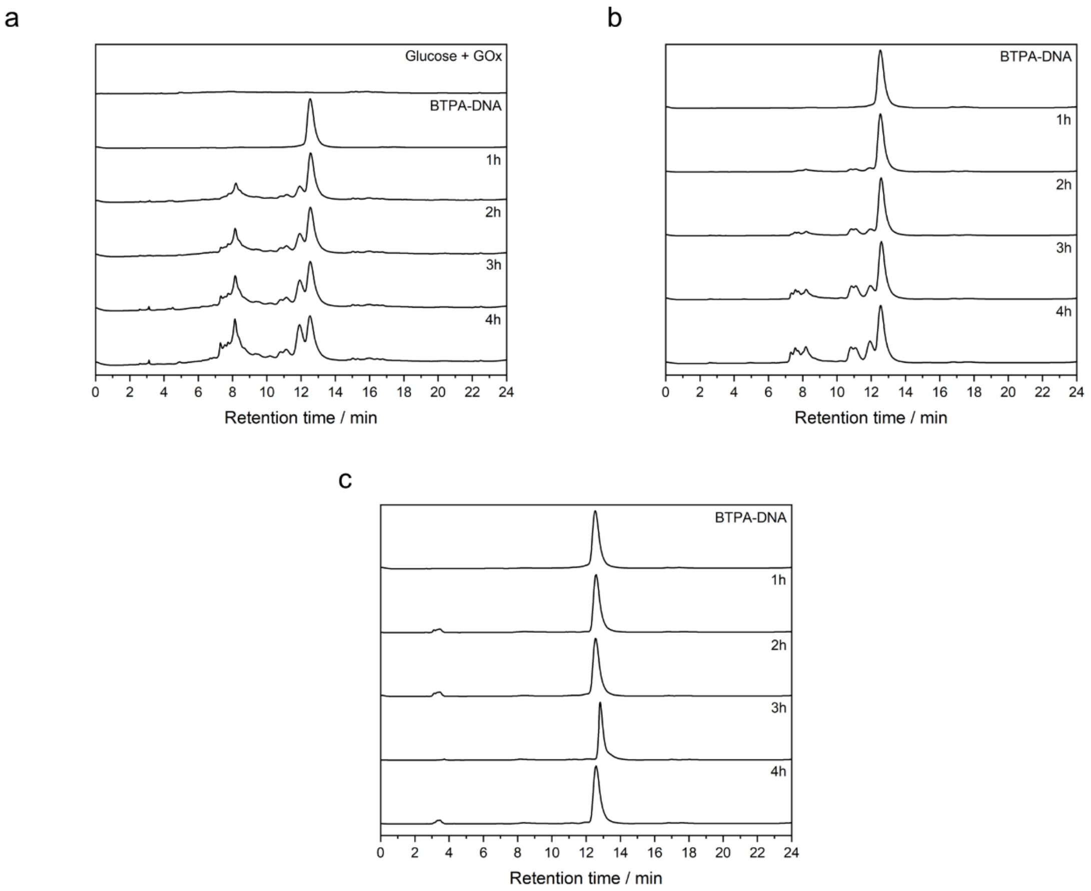


**Figure S4.** Degradation profiles of BTPA-DNA as measured by aqueous HPLC. BTPA-DNA was treated under the polymerization conditions without (a) and with sodium pyruvate at 50 mM (b) and 200 mM (c) in the absence of monomer and initiator. Significant decomposition of the BTPA-DNA occurred, when no pyruvate was present (a). In contrast, when pyruvate was added, the decomposition could be largely suppressed (b) or even entirely alleviated (c).

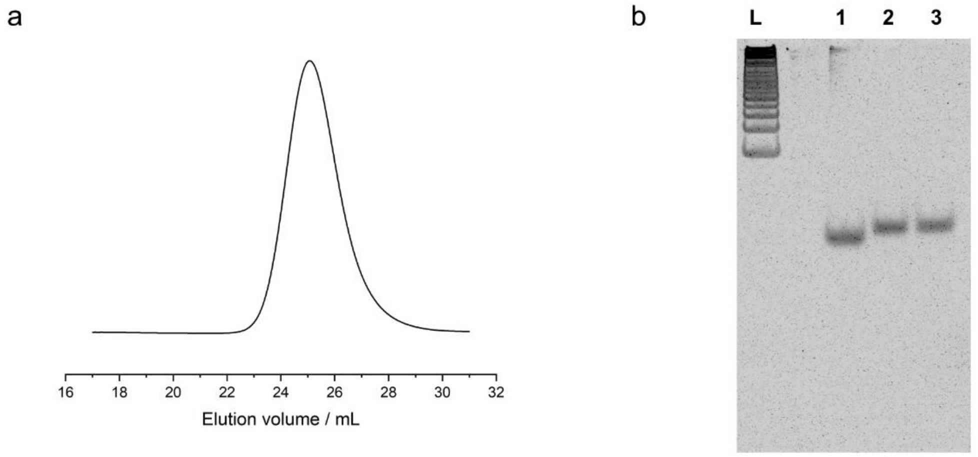


**Figure S5.** Stability test of DNA during RAFT polymerization of DMA ([DMA]:[BTPA] = 375:1, [BTPA] = 2 mM) under the employed enzyme degassing conditions as monitored by 10 % native PAGE. The polymerization was conducted in the presence of a Rho6G-DNA sequence (Rh6G – 5' – T TTT CTC TAC CAC CTA CTA — 3') at 25 $\mu$M. a) GPC trace of the polymer obtained after polymerization. b) Monitoring of the DNA stability during RAFT polymerization by 10 % native PAGE. L, DNA ladder; lane 1, Rho6G-DNA; lane 2, Rho6G-DNA after RAFT polymerization for 2 hours; lane 3, Rho6G-DNA after RAFT polymerization for 4 h.

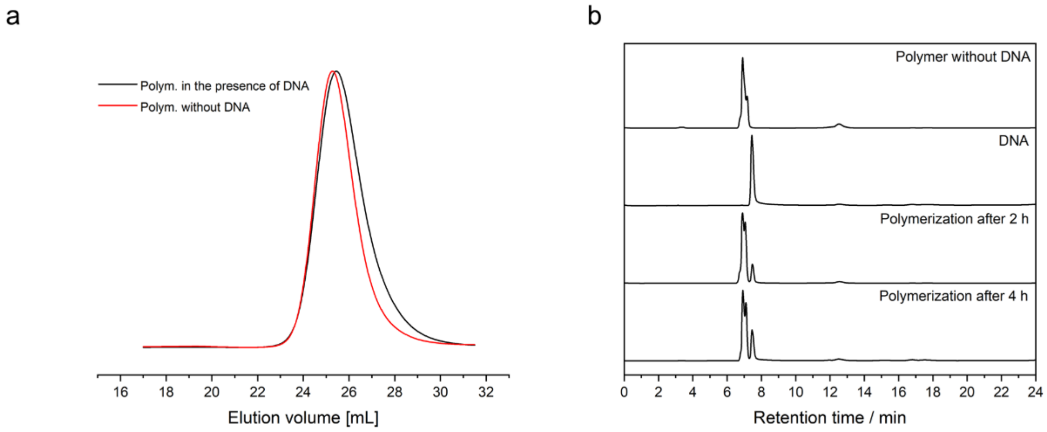


**Figure S6.** Stability test of DNA during RAFT polymerization of DMA ([DMA]:[BTPA] = 200:1, [BTPA] = 2 mM) under the employed enzyme degassing conditions as monitored by HPLC. The polymerization was conducted in the presence of an unfunctional DNA sequence (5' – T TTT CTC TAC CAC CTA CTA – 3') at 500 $\mu$M a) GPC traces of the polymers obtained in the presence and in the absence of the single-stranded DNA sequence, demonstrating that the presence of DNA does not adversely affect the polymerizations. b) Monitoring of the DNA stability during RAFT polymerization by aqueous HPLC, where no additional peaks were detected after polymerization, indicating that the DNA sequence remains intact and unaffected by the polymerization.

**Table S3.** Homopolymerizations from BTPA via thermal RAFT polymerization under ambient conditions for DNA-polymer mixing experiments.

| Entry | Monomer | [M]:[BTPA-DNA]:[I] [a] | Time (h) | Conversion [b] (%) | $M_{n,\ theo.}$ [c] (kDa) | $M_{n,\ app.}$ [d] (kDa) | $Đ$ [d] |
|---|---|---|---|---|---|---|---|
| RP1 | DMA | 400:1:0.1 | 4 | 82 | 32.8 | 32.0 | 1.28 |
| RP2 | DMA | 200:1:0.1 | 4 | 89 | 17.9 | 14.4 | 1.31 |
| RP3 | NAM | 400:1:0.1 | 4 | 96 | 54.4 | 34.5 | 1.36 |
| RP4 | NAM | 200:1:0.1 | 4 | 91 | 25.9 | 20.6 | 1.23 |
| RP5 | HEA | 200:1:0.1 | 4 | 89 | 20.9 | 25.4 | 1.32 |
| RP6 | OEGA | 40:1:0.1 | 4 | 90 | 17.5 | 12.4 | 1.28 |

[a] Polymerizations were conducted at 45 °C in DPBS/*t*BuOH (85/15, v/v) using [BTPA] = 5 mM and enzyme degassing at [glucose] = 100 mM, [GOx] = 1 $\mu$M and [SP] = 50 mM. [b] Conversions were determined by $^1$H NMR in $D_2O$ using DMF (2.5%) as internal standard. [c] Theoretical molecular weights were calculated based on the following equation: $M_{n,\ theo.} = [M]_0/[DNA]_0 \times MW^M \times conv.(NMR) + MW^{DNA}$. [d] Apparent molecular weights and dispersity values were determined by GPC with DMF as the eluent using PMMA calibration standards without correction.

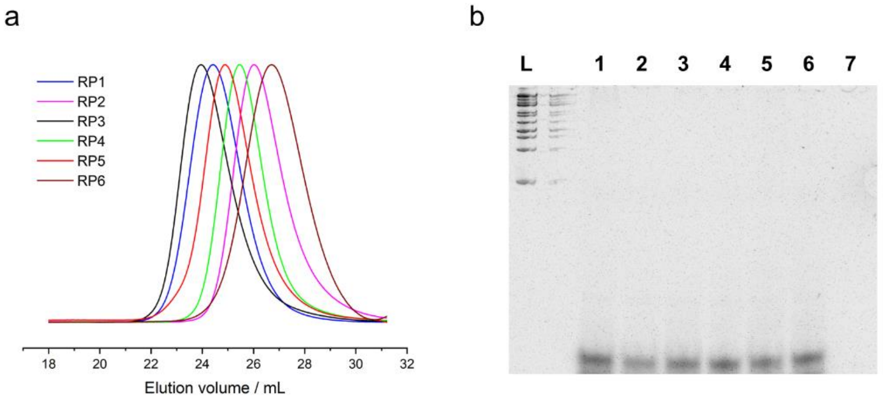


**Figure S7**. DNA-polymer mixing experiments to exclude unspecific binding of the DNA and the synthetic polymer due to physical entanglement. The polymerizations were conducted as depicted in Table S3. Each polymer was subsequently incubated with an amine-terminated single-stranded DNA sequence ($NH_2$ – 5’ – T TTT CTC TAC CAC CTA CTA – 3’) under the polymerization conditions prior to PAGE analysis. a) GPC traces of the polymers synthesized for the DNA-polymer mixing experiments. b) Gel electrophoresis after physical mixing of the polymers with an amine-terminated DNA sequence. Lane 1. Poly(DMA) (DPn = 400), Lane 2. Poly(DMA) (DPn = 200), Lane 3. Poly(NAM) (DPn = 400), Lane 4. Poly(NAM) (DPn = 200), Lane 5. Poly(HEA) (DPn = 200), Lane 6. Poly(OEGA) (DPn = 40), Lane 7: Polyethyleneimine.

**Table S4.** Synthesis of a rhodamine B-containing DNA-polymer conjugate.

| Entry | Monomer | [M]:[BTPA]:[I] a | Time (h) | [BTPA] (mM) | Conversion b (%) | $M_{n, theo.}$ c (kDa) | $M_{n, app.}$ d (kDa) | $Đ$ d |
|---|---|---|---|---|---|---|---|---|
| FP1 | HEA-co-RA | 249:1:1:0.1 | 4 | 2 | 64 | 25.0 | 25.2 | 1.27 |

[a] Polymerization was conducted at 50 °C in DPBS/*t*BuOH (85/15, v/v) using enzyme degassing at [glucose] = 100 mM, [GOx] = 1 $\mu$M and [SP] = 50 mM. [b] Conversions were determined by $^1$H NMR in $D_2O$ using DMF (2.5%) as internal standard. [c] Theoretical molecular weights were calculated based on the following equation: $M_{n, theo.} = [HEA]_0/[DNA]_0 \times MW^{HEA} \times$ conv.(NMR) + $[RA]_0/[DNA]_0 \times MW^{RA} \times$ conv.(NMR) + $MW^{DNA}$. [d] Apparent molecular weights and dispersity values were determined by GPC with DMF as the eluent using PMMA calibration standards without correction.

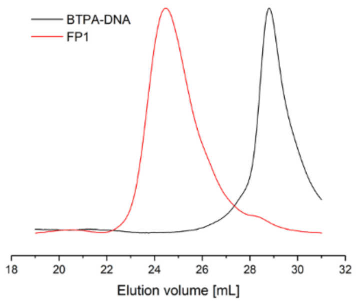


**Figure S8.** GPC traces of BTPA-DNA (black line) and **FP1** (red line) as measured by DMF GPC using PMMA calibration standards.

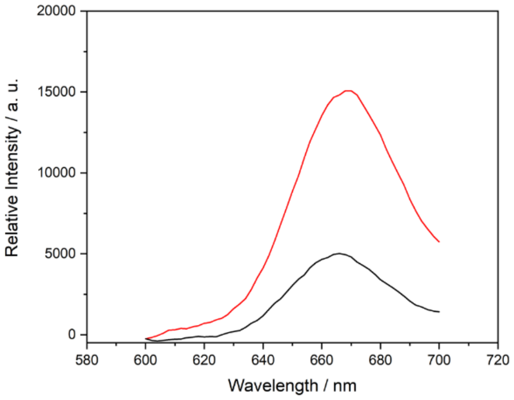


**Figure S9.** Emission spectra of **FP1** after hybridization with matching Cy5-DNA (red line) and mismatching Cy5-DNA (black line), respectively, upon excitation with an excitation filter at 485/20 nm. The spectra were obtained by subtracting the obtained emission spectrum after hybridization by the initial spectra of **FP1** and Cy5-DNA alone, resulting in the Cy5 emission caused by FRET.

**Table S5.** Synthesis of DNA-diblock copolymer conjugates.

| Entry | Monomer | [M]:[BTPA]:[I] a | Time (h) | [BTPA] (mM) | Conversion b (%) | $M_{n, theo.}$ c (kDa) | $M_{n, app.}$ d (kDa) | $Đ$ d |
|---|---|---|---|---|---|---|---|---|
| DP1.1 | DMA | 125:1:0.2 | 2 | 2 | 93 | 17.6 | 12.4 | 1.26 |
| DP1.2 | NAM | 125:1:0.2 | 2 | 1 | <10 | - | 15.5 | 1.47 |
| DP2.1 | DMA | 125:1:0.2 | 2 | 2 | 90 | 17.2 | 12.5 | 1.26 |
| DP2.2 | NAM | 125:1:0.2 | 2 | 1 | >95 | 36.1 | 33.0 | 1.34 |

[a] Polymerizations were conducted at 50 °C in DPBS/*t*BuOH (85/15, v/v) using enzyme degassing at [glucose] = 100 mM, [GOx] = 1 $\mu$M and [SP] = 50 mM. [b] Conversions were determined by $^1$H NMR in $D_2O$ using DMF (2.5%) as internal standard. [c] [c]Theoretical molecular weights were calculated based on the following equation: $M_{n, theo.} = [M]_0/[DNA]_0 \times MW^{M} \times$ conv.(NMR) + $MW^{DNA}$. [d] Apparent molecular weights and dispersity values were determined by GPC with DMF as the eluent using PMMA calibration standards without correction.

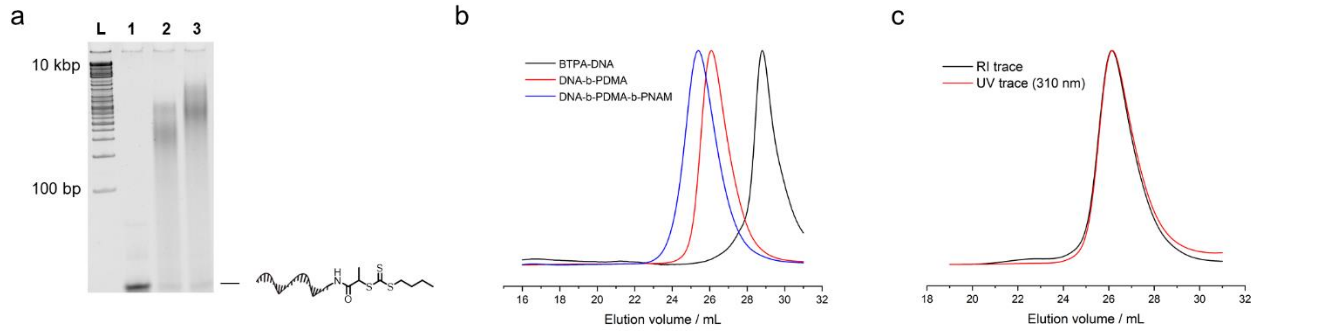


**Figure S10**. (a) BTPA-DNA and the DNA-polymer conjugates **DP1.1** and **DP1.2** analyzed by 20 % native PAGE. L, DNA ladder; lane 1, BTPA-DNA; lane 2. **DP1.1**; lane 3. **DP1.2**. (b) GPC traces of BTPA-DNA (black line), **DP1.1** (red line) and **DP1.2** (blue line) as measured by DMF GPC with PMMA calibration standards. (c) Overlay of the RI trace (black line) and the UV trace at $\lambda$ = 310 nm (red line) after polymerization of the first polymer block from DNA as measured by DMF GPC using PMMA calibration standards.

**Table S6.** Polymerization-induced self-assembly from single stranded DNA.

| Entry | Monomer | [M]:[BTPA]:[I] [a] | Time (h) | [BTPA] (mM) | Conversion [b] (%) | $M_{n,\,theo.}$ [c] (kDa) | $M_{n,\,app.}$ [d] (kDa) | $Đ$ [d] |
|---|---|---|---|---|---|---|---|---|
| PISA1 | DAAm-co-DMA | 40:10:1:0.2 | 4 | 2 | >95 | 13.8 | 9.1 | 1.26 |
| PISA2 | DAAm-co-DMA | 80:20:1:0.2 | 4 | 2 | >95 | 21.6 | 14.7 | 1.29 |
| PISA3 | DAAm-co-DMA | 160:40:1:0.2 | 4 | 1.5 | >95 | 37.1 | 27.4 | 2.42 |
| PISA4 | DAAm-co-DMA | 200:50:1:0.2 | 4 | 1.2 | >95 | 44.8 | 20.5 | 1.47 |

[a] Polymerizations were conducted at 50 °C in DPBS using enzyme degassing at [glucose] = 100 mM, [GOx] = 1 $\mu$M and [SP] = 50 mM. [b] Conversions were determined by $^{1}$H NMR in $D_2O$ using DMF (2.5 %) as internal standard. [c] Theoretical molecular weights were calculated based on the following equation: $M_{n,\,theo.} = [DAAm]_0/[DNA]_0 \times MW^{DAAm} \times conv.(NMR) + [DMA]_0/[DNA]_0 \times MW^{DMA} \times conv.(NMR) + MW^{DNA}$. [d] Apparent molecular weights and dispersity values were determined by GPC with DMF as the eluent using PMMA calibration standards without correction.

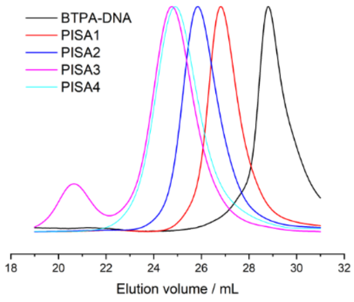


**Figure S11**. GPC traces of BTPA-DNA (black line) and the DNA-polymer conjugates **PISA1-PISA4** (coloured lines) as measured by DMF GPC with PMMA calibration standards.

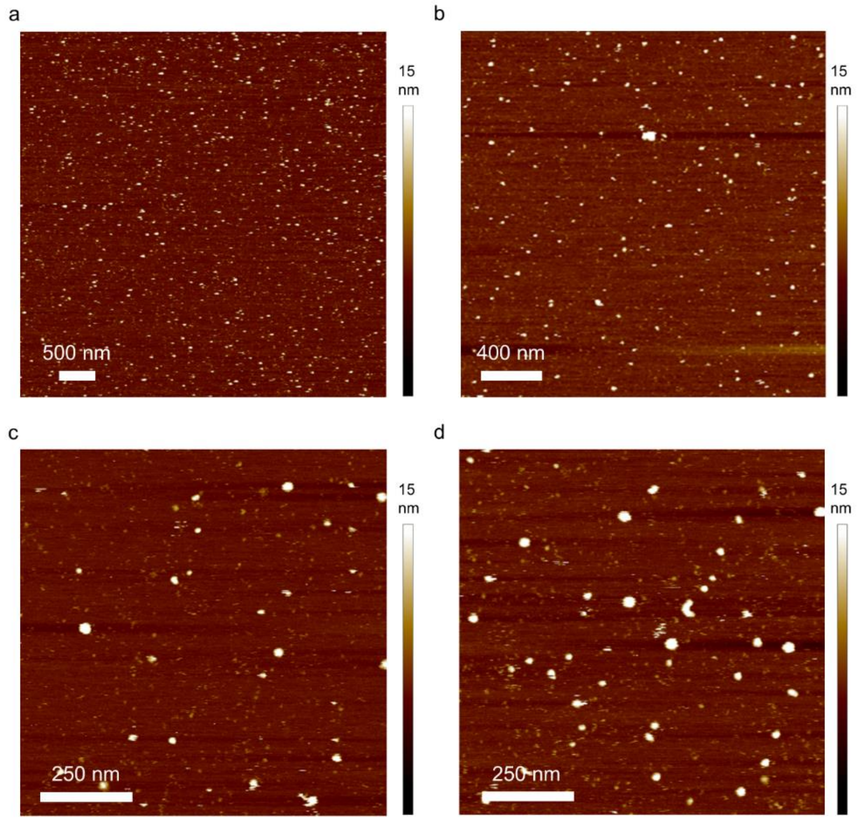


**Figure S12.** AFM images of DNA-polymer micelles as synthesized by RAFT polymerization from BTPA-DNA using a [DAAM]:[DMA] ratio of 80:20 and a [M]:[BTPA-DNA] ratio of 50:1.

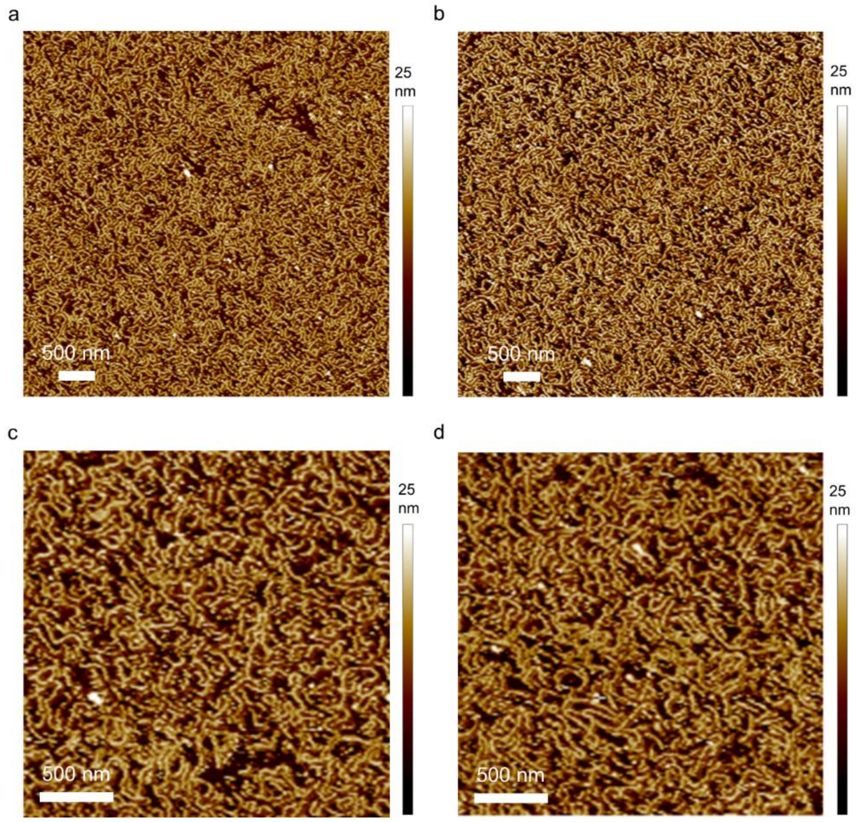


**Figure S13.** AFM images of DNA-polymer worms as synthesized by RAFT polymerization from BTPA-DNA using a [DAAM]:[DMA] ratio of 80:20 and a [M]:[BTPA-DNA] ratio of 100:1.

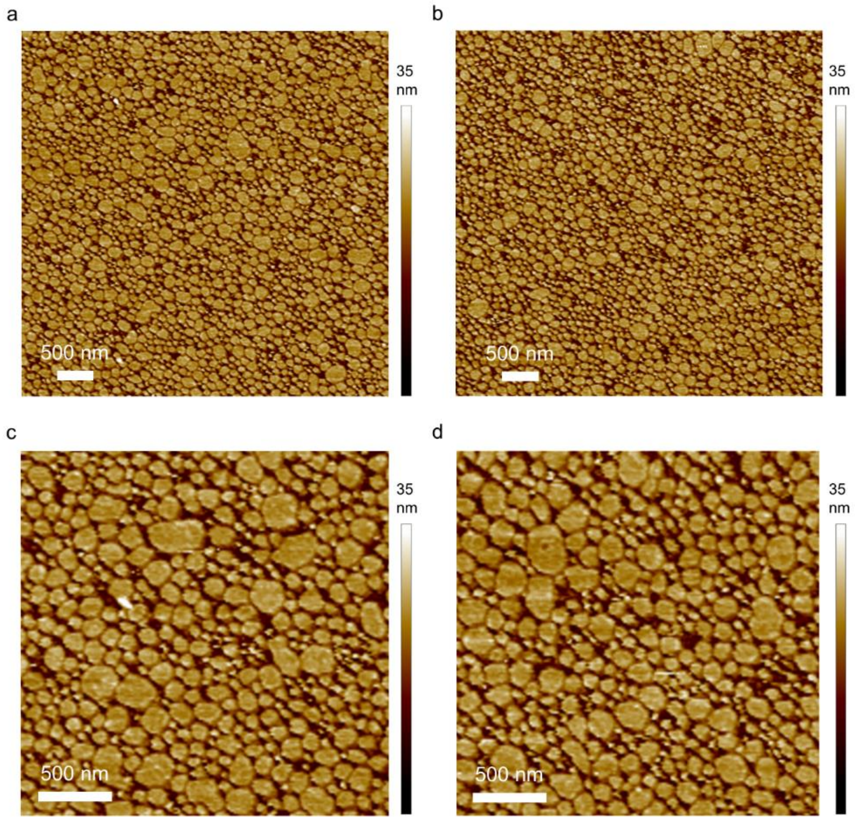


**Figure S14.** AFM images of DNA-polymer discs as synthesized by RAFT polymerization from BTPA-DNA using a [DAAM]:[DMA] ratio of 80:20 and a [M]:[BTPA-DNA] ratio of 200:1.

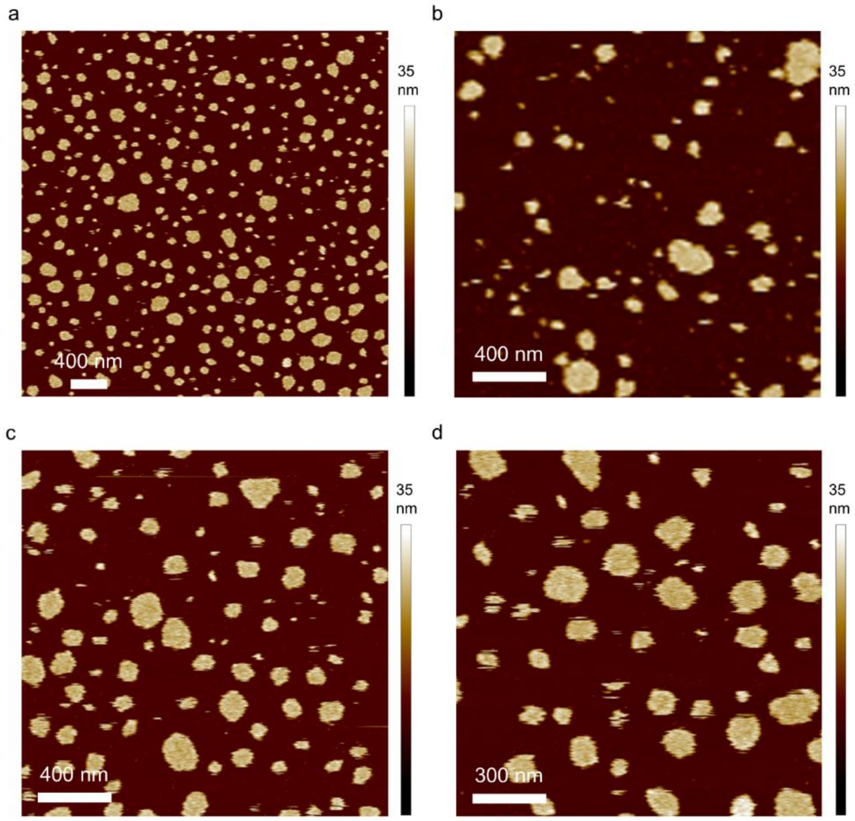


**Figure S15.** AFM images of DNA-polymer discs as synthesized by RAFT polymerization from BTPA-DNA using a [DAAM]:[DMA] ratio of 80:20 and a [M]:[BTPA-DNA] ratio of 250:1.

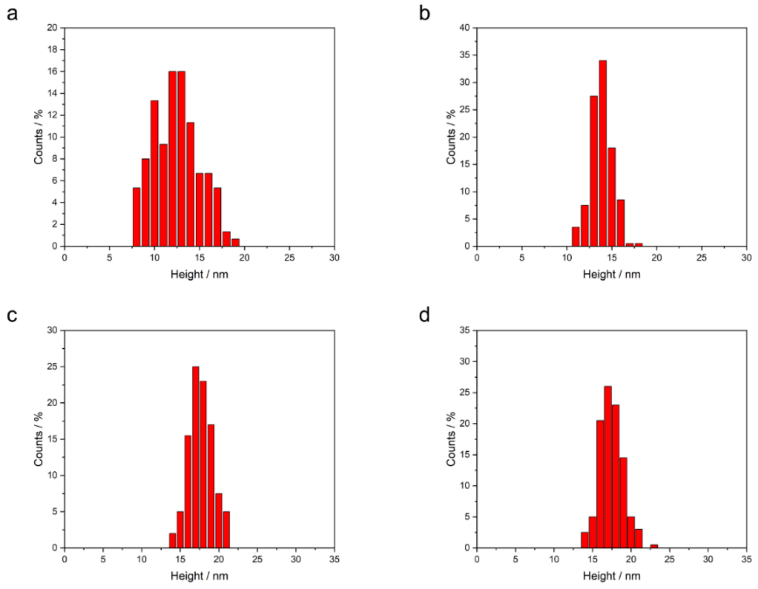


**Figure S16**. Height histograms of the DNA-polymer nanostructures synthesized by polymerization-induced self-assembly (PISA) from DNA using *N*,*N*-dimethylacrylamide (DMA) and diacetone acrylamide (DAAm) as the monomers. Different nanostructures were synthesized by targeting different degrees of polymerization ($DP_n$) and a height distribution histogram was made for each targeted $DP_n$: (a) $DP_n$ = 50. (b) $DP_n$ = 100. (c) $DP_n$ = 200. (d) $DP_n$ = 250. The heights of the nanostructures were determined from AFM by height profile analysis and the height distribution histograms were then created by counting the heights of at least 200 individual nanostructures.

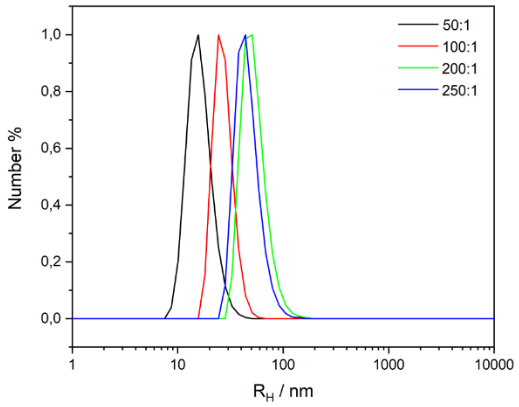


**Figure S17**. DLS data of the synthesized DNA-polymer nanostructures. Each of the three morphologies demonstrates a characteristic signal in DLS, where the mean diameter of the micelles, worms and disc aggregates were measured to be 16.5 ± 1.5 nm, 27.3 ± 0.6 nm and 53.7 ± 0.9 nm, respectively.

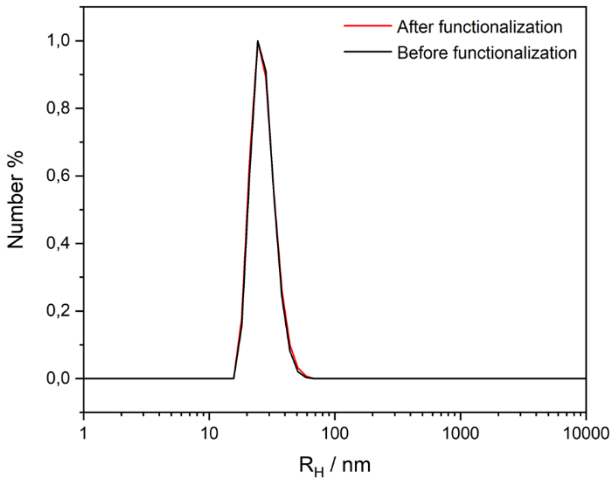


**Figure S18.** DLS of the DNA-polymer worms before (black line) and after functionalization (red line) with a complementary Rh6G-DNA sequence. The DLS spectra remain unchanged after hybridizing a small fraction of a complementary Rh6G-DNA sequence to the DNA-polymer nanostructure.

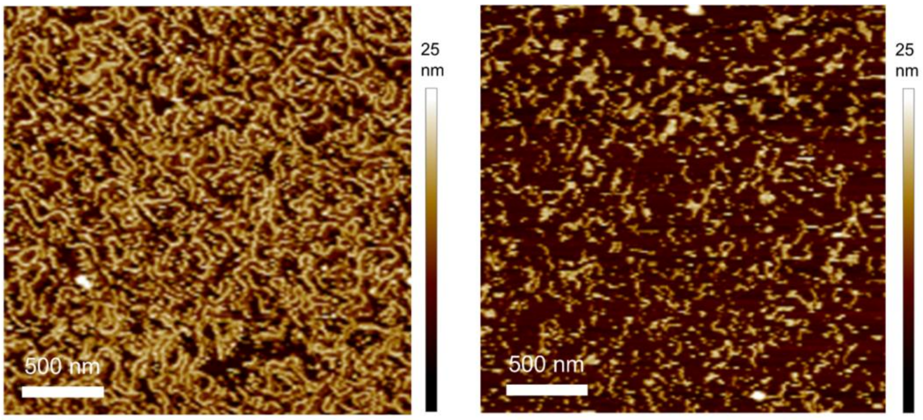


**Figure S19.** AFM images of the DNA-polymer worms before (left) and after functionalization (right) with the complementary Rh6G-terminated DNA sequence. A moderate change in uniformity of the structures could be seen by AFM after hybridization, however, their elongated morphologies were maintained.

## References

[1] T. Lueckerath, T. Strauch, K. Koynov, C. Barner-Kowollik, D. Y. W. Ng, T. Weil, *Biomacromolecules* **2019**, *20*, 212.
[2] X. Han, E. Wang, Y. Cui, Y. Lin, H. Chen, R. An, X. Liang, M. Komiyama, *Electrophoresis* **2019**, *40*, 1708.
[3] M. R. Green, J. Sambrook, *Cold Spring Harbor protocols* **2019**, *2019*.
[4] C. A. Schneider, W. S. Rasband, K. W. Eliceiri, *Nature methods* **2012**, *9*, 671.

## Author Contributions

T. L. conducted all of the experiments and characterization. K. K. measured FCS. S. L. and A. W. contributed the cryoTEM measurements. C. W. contributed to the PAGE analysis. L. N. helped with the DLS measurements. The project was supervised by C. B.-K., D.Y.W .N. and T. W. All authors contributed to writing the manuscript.
